# Bayesian spatio-temporal conditional autoregressive localized modeling techniques for socioeconomic factors and stunting in Indonesia

**DOI:** 10.1016/j.mex.2025.103464

**Published:** 2025-06-24

**Authors:** Aswi Aswi, Septian Rahardiantoro, Anang Kurnia, Bagus Sartono, Dian Handayani, Nurwan Nurwan

**Affiliations:** a Statistics Department, Universitas Negeri Makassar, Makassar, Indonesia; b IPB University, Bogor, Indonesia; c Universitas Negeri Jakarta, Jakarta Indonesia

**Keywords:** Bayesian, CARBayesST, Relative Risk, Spatio-temporal CAR, Stunting, Bayesian Spatio-temporal Conditional Autoregressive Localized Modeling

## Abstract

Stunting remains a persistent public health issue in Indonesia, exhibiting significant spatial and temporal variation. To address this, we employed a hierarchical Bayesian spatio-temporal localized Conditional Autoregressive (CAR) model that includes a clustering component to identify risk factors and estimate relative risk (RR) across 34 provinces from 2020 to 2022. A total of 480 models were evaluated, encompassing three variants of the Bayesian spatio-temporal localized CAR model, 32 covariate combinations, and five hyperprior settings. Assuming a Poisson likelihood for stunting counts, the optimal model was estimated using Markov Chain Monte Carlo methods and included two covariates, namely the poverty rate and the incidence of low birth weight, with up to five spatial clusters. Higher poverty levels and increased prevalence of low birth weight were significantly associated with elevated stunting risk among children under five. Spatio-temporal clustering patterns and the estimated relative risks of stunting varied across Indonesian provinces from 2020 to 2022. Nusa Tenggara Timur consistently ranked among the top three provinces with the highest risk (RR = 2.421 in 2020; 2.384 in 2021; 2.676 in 2022). The highest risk was observed in Sulawesi Barat in 2022 (RR = 2.768), while DKI Jakarta consistently showed the lowest (RR = 0.004).

Some key points of the article are:

• Bayesian spatio-temporal models facilitate the classification of distinct area groups
• The models were employed to analyze stunting patterns in Indonesia.
• The inclusion of covariates influenced the number of groups identified.

## Graphical abstract

**Figure:**
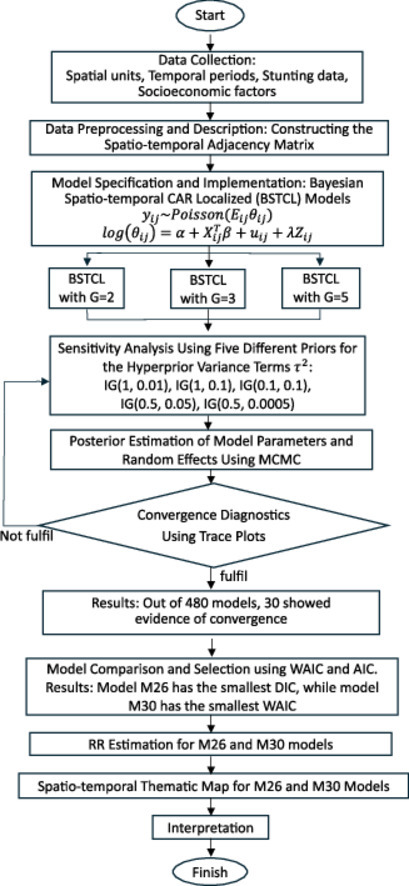

Specifications table

**Table:** 

Subject area:	Mathematics and Statistics
More specific subject area:	Statistics: Bayesian, spatio-temporal modeling
Name of your method:	Bayesian Spatio-temporal Conditional Autoregressive Localized Modeling
Name and reference of original method:	D. Lee, A. Rushworth, and G. Napier, Spatio-Temporal Areal Unit Modeling in R with Conditional Autoregressive Priors Using the CARBayesST Package, J. Stat. Softw. 84 (2018), pp. 1–39. D. Lee and A. Lawson, Quantifying the Spatial Inequality and Temporal Trends in Maternal Smoking Rates in Glasgow, Ann. Appl. Stat. 10 (2016), pp. 1427–1446.
Resource availability:	The data and R code will be made available upon request

## Background

Stunting is a condition observed in children characterized by a failure to reach expected height standards for their age. Stunting remains a significant public health issue in developing nations, with varying prevalence both within and among countries [1]. The condition typically arises due to chronic malnutrition, illnesses, or environmental factors leading to intestinal inflammation [2,3]. Recent studies have examined stunting and its determinants using spatial modeling approaches. A Bayesian geostatistical model with the R-Integrated Nested Laplace Approximation (R-INLA) package has been employed to estimate and spatially map the prevalence of stunting among children under five in Ghana, aiming to identify communities at heightened risk [4]. Similarly, a Bayesian distributional regression has been utilized to analyze stunting distribution in Zambia, uncovering a significant non-linear relationship between maternal education, household wealth, and stunting [5]. They found that an increase in maternal education beyond the eighth grade was associated with a reduction in stunting prevalence. These studies, however, consider only the spatial component and were conducted outside Indonesia. In Indonesia, Bayesian spatial Conditional Autoregressive (CAR) models have previously been used to investigate stunting [6–8]. They found that the incidence of stunting is positively influenced by the percentage of poverty and the rate of malnutrition among children aged 0–59 months. As the levels of poverty and malnutrition in this age group increase within a region, the risk of stunting also rises accordingly [7]. However, their study only focused on one province in Indonesia [7].

Bayesian spatial or spatio-temporal models are valuable tools for modeling stunting. However, only a few studies incorporated Bayesian spatio-temporal random effects in their stunting models. Bayesian hierarchical spatio-temporal regression using R-INLA has been employed to determine the independent effects of conflict on wasting and stunting in children aged 6–59 months across Somalia [9]. Another study utilized a spatio-temporal Bernoulli regression model to investigate the predictors of wasting, stunting, and lower upper arm circumference among children aged 6–59 months in Somalia. This study, based on a cross-sectional household survey, aimed to inform more comprehensive nutrition interventions [2]. Additionally, Bayesian space-time geostatistical models using R-INLA were used to estimate the prevalence and distribution of stunting among children under five in Somalia from 2007 to 2010, exploring the role of environmental covariates in this forecast [3]. Bayesian spatio-temporal geoadditive semiparametric models have also been used to examine the spatio-temporal clustering and the socio-demographic and socio-ecological factors associated with district-level spatial patterns of childhood stunting in Ghana. The findings indicated that childhood stunting in Ghana is not randomly distributed spatially but rather clustered [10]. This study further introduces three variants of the Bayesian spatio-temporal Conditional Autoregressive (ST CAR) localized model to better capture discontinuities in regional clustering. The ST CAR localized model offers enhanced flexibility in detecting local discontinuities and heterogeneity in spatial patterns, which is particularly valuable in a diverse setting like Indonesia. Based on the author's research, there has been no published application of the Bayesian ST CAR model in addressing stunting cases in Indonesia. This highlights an important methodological gap. To the best of the authors’ knowledge, this is the first study to implement a Bayesian spatio-temporal CAR localized model to analyze stunting across all 34 provinces in Indonesia. This allows for a more thorough assessment of spatial clustering and temporal dynamics in stunting prevalence across the country.

Focusing solely on the spatial aspect of diseases can highlight regions with increased or decreased risk but fails to account for the temporal fluctuations of risk, which can be equally or more significant. Given that stunting varies across different regions and over time, it is essential to consider both spatial and temporal components. Bayesian methods are advantageous due to their flexibility in incorporating additional information, such as temporal and spatial structures, via prior distributions. Beyond the ability to incorporate prior information, Bayesian methods offer several important advantages over frequentist approaches, particularly in the context of spatio-temporal modeling. These include full probabilistic inference, which allows for direct estimation of uncertainty through posterior distributions, and the capacity to seamlessly integrate multiple data sources or expert knowledge. Such strengths make Bayesian approaches especially valuable in public health research, where data may be sparse, heterogeneous, or derived from various levels of information. Therefore, this study aims to evaluate the associations between socioeconomic factors and stunting incidence as well as to estimate the relative risk (RR) of stunting across 34 geographic areas in Indonesia from 2020 to 2022. This will be achieved using three variants of the Bayesian ST CAR localized model. The main contributions of this study are threefold: (1) the introduction of a spatio-temporal CAR modeling framework for nationwide stunting analysis in Indonesia, (2) the identification of associations between stunting and socioeconomic determinants over time and space, and (3) the provision of area-specific relative risk estimates that can inform better-targeted policy interventions. The remainder of the manuscript is structured as follows: Section 2 describes the data and methodology, Section 3 presents the results, Section 4 discusses the findings, and Section 5 concludes the study.

## Method details

### Data

Data on the number of toddlers and stunted toddlers by province from 2020 to 2022 were sourced from the Directorate General of Regional Development, Ministry of Home Affairs [11] official website. The study's covariates are detailed in Table 1.

**Table 1:** The study's covariates used in this study.

	Variables	Operational definition of variables
X_1_	Poverty	The percentage of poor people is calculated by dividing the number of poor people by the total population in the same time period, and then multiplying by 100 to get the percentage [12].
X_2_	Exclusive Breastfeeding	The percentage of babies aged <6 months who receive exclusive breastfeeding [13].
X_3_	Low Birth Weight	The percentage of mothers who gave birth to live-born children in the past two years, where the most recent child had a low birth weight [13].
X_4_	Immunization	The percentage of 12- to 23-month-old children who received full basic immunization [13]
X_5_	Have Received Breast Milk.	The proportion of children aged 0–23 months who have ever received breast milk [13].
X_6_	Still Being Breastfed	The percentage of children aged 0–23 months who have been and are still being breastfed [13].
X_7_	Child Diet Diversity	The percentage of children aged 6–23 months who consume at least five of eight food and drink groups daily [13].

Before running models, we evaluated the correlation among covariates using Spearman’s correlation coefficient.

### Summary of related works

Summary of related works of stunting was given in Table 2.

**Table 2:** Summary of Related Works on Stunting (2020–2025).

References	Research Method	Aims	Limitations	Relevance to This Study
Niragire et al. 2025 [14]	A geo-additive binary logistic regression analyses	To examine the spatial and temporal variations in under-five child stunting rates and their associated factors in Rwanda from 2010 to 2020.	Does not consider Bayesian ST CAR	Provides insights into spatio-temporal modeling of stunting cases
Aswi et al., 2024 [15]	Bayesian spatial CAR	To assess the risk of stunting and influencing factors in Indonesia that include covariates	Does not consider temporal effects	Provides insights into spatial modeling of stunting cases
Rahardiantoro et al., 2024 [16]	The Modified Generalized Lasso	To identify factors associated with stunting in Indonesia using a Modified Generalized Lasso of Spatio-temporal modeling	Does not consider Bayesian ST CAR	Provides insights into spatio-temporal modeling of stunting cases
Azis & Aswi, 2023 [6]	Bayesian spatial clustering	To identify of spatial clustering of stunting cases in Indonesia using Bayesian spatial CAR	Does not consider temporal effects	Provides insights into spatial modeling of stunting cases
Uwiringiyimana et al., 2022 [1]	Bayesian geostatistical model	To map the spatial distribution of stunting prevalence and risk factors in Rwanda.	Does not consider temporal effects	Provides methodological basis for spatial mapping of stunting
Aswi & Sukarna, 2022 [7]	Bayesian spatial modeling	To identify factors affecting stunting in South Sulawesi	Focuses on a single province in Indonesia	Foundational study for national-level spatial analysis
Amoako Johnson, 2022 [17]	Bayesian spatio-temporal geoadditive model	To identify spatio-temporal clustering patterns and examine socio-demographic factors influencing stunting in Ghana.	Does not consider Bayesian ST CAR	Demonstrates the value of spatio-temporal approaches for this study
Moonga et al., 2021 [5]	Bayesian distributional regression	To model the nonlinear relationships between maternal education, household wealth, and stunting in Zambia	Does not include temporal effects	Supports distributional regression approach for determinant analysis
Aheto & Dagne, 2021 [4]	Bayesian geostatistical model	To identify communities at higher risk where interventions and further research can be targeted.	Does not include temporal effects	Geostatistical approach for mapping stunting risk
Hossain & Khan, 2020 [18]	A hierarchical Bayesian spatial logistic model	To assess the association between household livestock ownership and childhood stunting, and to examine geospatial variations at the district level in Bangladesh	Does not include temporal effects	Provides methodological basis for spatial mapping of stunting

### Problem statement

Stunting remains a persistent public health challenge in Indonesia, with significant spatial and temporal variation observed across its provinces. Traditional spatial modeling approaches have been employed to identify high-risk regions; however, these often overlook the temporal dynamics and localized clustering patterns that can influence the effectiveness of interventions. Moreover, while Bayesian spatial and spatio-temporal models have been widely utilized in the analysis of stunting, several critical gaps remain unaddressed. First, existing studies primarily focus on specific regions within Indonesia, lacking a comprehensive nationwide assessment. Second, many previous analyzes emphasize spatial relationships but fail to incorporate temporal dynamics, limiting their ability to capture changes over time. Third, no prior research has employed the Bayesian spatio-temporal CAR localized model to detect regional discontinuities in stunting prevalence. To address these gaps, this study applies the Bayesian spatio-temporal CAR localized model, enabling a more precise evaluation of stunting patterns across all 34 provinces in Indonesia by integrating both spatial clustering and temporal trends. To formally represent the research objective, we consider the following mathematical formulation.

### Model formulation

Three variants of the Bayesian ST CAR localized models were utilized to evaluate the relative risk and whether this formed groups, as well as the significance of socioeconomic variables on stunting throughout Indonesia. It assumes the response variable ($y_{ij})\;$follows a Poisson distribution and is formulated as follows:

(1) $$y_{ij}∼\text{Poisson}\left(E_{ij}\theta_{ij}\right)\;$$

(2) $$log\left(\theta_{ij}\right)=\alpha +\;x_{ij}^{T}\beta +\psi_{ij}$$

where $y_{ij}\;$represents the observed number of stunting cases in the *i*th area and *j*th time, *i* = 1, 2, 3, 4, …, 34; *j* = 1, 2, 3; and $E_{ij}$ and $\theta_{ij}$ denote the expected number of stunting cases and the relative risk of stunting cases respectively, each in area *i* at time *j*. The expected number of stunting cases ($E_{ij})$ is determined by multiplying the overall incidence rate for the entire Indonesia region throughout the study period by the at-risk population in each area and time, calculated as:

(3) $$E_{ij}=\frac{\sum_{i}\sum_{j}y_{ij}}{\sum_{i}\sum_{j}n_{ij}}n_{ij}.$$

The variable $n_{ij}$ represents the number of toddlers aged 0–59 months whose height was measured in each district/city *i* = 1, 2, 3, …, 34 and time *j* = 1, 2, 3.

The parameter α represents the overall level of relative risk, while $\psi_{ij}$ denotes the latent component for area *i* and time *j* encompassing one or more sets of spatio-temporally autocorrelated random effects. $x_{ij}^{T}$ represents the transpose of the covariate vector, forming a linear combination of the selected covariates (fixed effects). The regression parameters **β** for the covariates are assigned a multivariate Gaussian prior with weakly informative hyperparameters. All models were computed using Markov chain Monte Carlo (MCMC) methods through the CARBayesST package version 4.0 [19] in R software version 4.3.2 [20].

### Spatio-temporal CAR localized model

The spatio-temporal CAR localized model proposed by Lee and Lawson [21] can detect clusters of regions that exhibit distinct response values compared to their geographical and temporal neighbors. Unlike models that assume neighboring areas will have similar responses after considering certain covariates, this model does not have that restriction. Its formulation is presented as follows:

(4) $$\psi_{ij}\;=\;u_{ij}+\;\lambda_{Z_{ij}},$$

The two sets of latent components, $u_{ij}\;$and $\lambda_{Z_{ij}}$ are smoothing components representing spatially and temporally autocorrelated variation and a piecewise constant grouping or intercept component, respectively.

Spatially and temporally adjacent data points (*y_ij_, y_kl_*) will be autocorrelated if they share the same intercept $\lambda_{Z_{ij}}=\lambda_{Z_{kl}}$. However, they can exhibit very different values if their intercepts differ, $\lambda_{Z_{\mathrm{ij}}}\neq \lambda_{Z_{\mathrm{kl}}}$. In the context of stunting, differing intercepts allow neighboring areas in both space and time to have significantly different probabilities of stunting, so the piecewise intercept can identify distinct groups of areas with varying probabilities of stunting. These groups, ordered by their intercepts, form up to *G* distinct categories ($\lambda_{1}<\;\lambda_{2}<\;\lambda_{3}<\ldots <\lambda_{G}$), as determined by the prior

(5) $$\begin{array}{c} \lambda_{k}∼\;\mathrm{Unif}\mathrm{orm}\left(\lambda_{k-1},\lambda_{k+1}\right)\;\mathrm{for}\;k=1,\;2,\cdots ,\;G \end{array}$$

where λ_0_ = -∞ and λ*_G_*_+__1_ = +∞. An observation in area *i* at time *j* is assigned to one of the G intercepts by $Z_{ij}\in \;\left\{1,\;2,\;3,\;\ldots ,\;G\right\}.$ The value of *G* is fixed in the model, and it is recommended to set it to a small odd number [22] such as *G* = 3 or *G* = 5. Values of *G* = 2 were also considered; larger values were not explored due to the limited number of spatial areas [23]. The value of $Z_{ij}$ is penalised towards the middle intercept value $G^{*}$: $G^{*}=\;\frac{G+1}{2}$ if G is odd and $G^{*}=\;\frac{G}{2}$ if G is even, through a penalty function $f(Z_{ij}):$

(6) $$f\left(Z_{ij}|Z_{i,j-1}\right)=\;\frac{\exp(-\delta \;\left[{\left(Z_{ij}-Z_{i,j-1}\right)}^{2}+{\left(Z_{ij}-G^{*}\right)}^{2}\right])}{\sum_{r=1}^{G}\exp\left(-\delta {\left(r-Z_{i,j-1}\right)}^{2}+\;{\left(r-G^{*}\right)}^{2}\right])}\text{for}\;j\;=\;2,\;\ldots ,J$$

$$f\left(Z_{i1}\right)=\;\frac{\exp(-\delta {\left(Z_{i1}-G^{*}\right)}^{2}]}{\sum_{r=1}^{G}\exp\left(-\delta {\left(r-G^{*}\right)}^{2}\right)}$$

The penalty parameter δ follow a uniform ditribution, δ∼ Uniform (L_δ_, U_δ_), where the hyperparameters L_δ_ and U_δ_ are set to 1 and 100, respectively, based on established recommendations [21].

The smoothing components $u_{ij}\;$are modelled using a multivariate autoregressive process with spatial autocorrelation $\rho_{S}=1$ which corresponds to an Intrinsic Conditional Autoregressive (ICAR) model, a widely used special case of the CAR model that induces strong spatial smoothing between neighboring areas.

$$u_{1}∼N\;\left(0,\;\tau^{2}Q{\left(W,\;\rho_{S}\right)}^{-1}\right),$$

$$\left(u_{j}|u_{j-1}\right)∼N\;\left(\rho_{T}u_{j-1},\;\;\tau^{2}Q{\left(W,\;\rho_{S}\right)}^{-1}\right)\;j\;=\;2,\;\cdots .J,$$

$$\tau^{2}∼\;\text{Inverse}-\text{Gamma}\;\left(1,\;0.01\right)$$

$$\rho_{T}\;∼\;\text{Uniform}\;\left(0,\;1\right)$$

These values were chosen for the case study in accordance with recommendations from Lee and Lawson [21]. A value of $\rho_{S}=1$ imposes rigorous spatial smoothing on $u_{j}$, ensuring that any abrupt changes (step changes) in the surface are primarily controlled by $\lambda_{Z_{ij}}$, while $\rho_{T}$ is the temporal autocorrelation parameter.

To evaluate the impact of priors on the estimation of the posterior distribution, we conducted a sensitivity analysis. Specifically, we tested five different priors for the hyperprior variance terms $\tau^{2}$: Inverse Gamma IG(1, 0.01), the default hyperprior specification in CARBayesST, IG(1, 0.1), IG(0.1, 0.1), IG(0.5, 0.05), and IG(0.5, 0.0005). The CARBayesST package facilitates the application of both univariate and multivariate spatio-temporal generalized linear mixed models for areal unit data, employing Bayesian inference through MCMC simulation.

### Comparing models

Three distinct model formulations (ST CAR localised with *G* = 2, *G* = 3, and *G* = 5) were each evaluated under five different prior specifications: IG(1, 0.01), IG(1, 0.1), IG(0.1, 0.1), IG(0.5, 0.05), and IG(0.5, 0.0005). Each formulation was further assessed using 32 covariate scenarios, including models both with and without covariates. This resulted in a total of 480 model combinations (3 × 5 × 32). Goodness-of-fit measures including the Deviance Information Criterion (DIC) [24], Watanabe-Akaike Information Criterion (WAIC) [25], group-specific coefficients, and the proportion of areas included in each group were used to compare models. Lower DIC and WAIC values, indicate better model fit. Model formulations and covariate combinations were further assessed by examining the 95 % posterior credible interval (CI), and considered significant if the CI does not include zero. Convergence checking is crucial in Bayesian spatio-temporal model analyzes using MCMC techniques. It involves assessing trace plots to ensure that Markov chains have reached a stationary distribution, thereby accurately representing the posterior distribution. Lack of convergence can lead to biased or inaccurate model inferences.

## Method validation

### Descriptive analysis

A descriptive analysis of the number of toddlers and stunted toddlers by province from 2020 to 2022 is presented in Table 3.

**Table 3:** A descriptive analysis of the number of toddlers and stunted toddlers.

	The number of toddlers			Stunting		
	2020	2021	2022	2020	2021	2022
Mean	215,259	415,565	464,611	23,457	39,457	38,859
95 % CI for Mean	90,649–339,869	178,718–652,411	231,525–697,696	10,195–36,718	17,910–61,003	19,767–57,952
5 % Trimmed Mean	153,400	310,337	362,797	17,665	30,264	31,410
Median	83,436	162,809	217,778	7862	15,454	17,888
Std. Deviation	357,134	678,804	668,026	38,007	61,751	54,719
Minimum	13,356	27,191	29,202	72	1007	3080
Maximum	1724,422	3149,244	3219,522	154,853	260,659	221,065
Range	1711,066	3122,053	3190,320	154,781	259,652	217,985
Interquartile Range	237,990	315,495	329,267	15,557	34,190	38,369
Skewness	3.21	2.92	2.95	2.53	2.59	2.41
Kurtosis	10.99	8.68	9.23	6.08	6.32	5.21

The Spearman correlations between stunting cases and the covariates shows that there is a substantial correlation between variables X_5_ and X_6_ (0.849), as well as a high correlation between X_2_ and X_6_ (0.607) and X_2_ and X_5_ (0.566). Due to these significant correlations, variables X_2_ and X_6_ were excluded from further analysis. Consequently, only five covariates are included in the spatial models.

### Bayesian spatio-temporal CAR

Three variants of the Bayesian spatio-temporal CAR localized model with *G* = 2, *G* = 3, and *G* = 5 were employed. These models were used to investigate the prevalence of stunting and examine its associations with selected covariates in Indonesia. We checked the convergence of the model using trace plots to ensure that the Markov chains have reached a stationary distribution. Out of 480 model combinations, only 30 converged successfully for *G* = 2, *G* = 3, and *G* = 5. From the 30 convergent models, the preferred model was selected based on the smallest DIC, WAIC, group-specific coefficients, and the proportion of areas included in each group (Table 4). The hyperpriors, covariates, DIC, WAIC, posterior quantities for covariates, and the number of areas per cluster for *G* = 2, *G* = 3, and *G* = 5 models are given in Table 4.

**Table 4:** The hyperpriors, covariates, DIC, WAIC, posterior quantities for covariates, and the number of areas per cluster for *G* = 2, *G* = 3, and *G* = 5.

*G* = 2											
Hyperprior		Model	DIC	WAIC	Posterior Quantities for Covariates		The number of areas per cluster				
					2.5 %	97.5 %	G1	G2			
IG(1, 0.01)	M1	Without Cov.	1663.519	3670.959	-	-	39	63			
	M2	X_1_ +	1644.542	3349.619	0.0187	0.0188	47	55			
		X_3_			0.0429	0.0431					
IG(1, 0.1)	M3	Without Cov.	1610.598	3332.633	-	-	38	64			
	M4	X_1_ +	1645.444	3678.797	0.0187	0.0188	47	55			
		X_3_			0.0427	0.043					
IG(0.1, 0.1)	M5	Without Cov.	1629.012	3487.865	-	-	38	64			
	M6	X_1_ +	1671.673	3898.311	0.0187	0.0187	47	55			
		X_3_			0.0429	0.0429					
IG(0.5, 0.05)	M7	Without Cov.	1670.676	3976.358	-	-	38	64			
	M8	X_1_ +	1683.864	3937.693	0.0186	0.0187	47	55			
		X_3_			0.0426	0.0429					
IG(0.5, 0.0005)	M9	Without Cov.	1632.381	3318.962	-	-	39	63			
	M10	X_1_ +	1659.206	3678.553	0.0187	0.0188	47	55			
		X_3_			0.0426	0.0429					

*G* = 3											
Hyperprior		Model	DIC	WAIC	Posterior Quantities for Covariates		The number of areas per cluster				
					2.5 %	97.5 %	G1	G2	G3		
IG(1, 0.01)	M11	Without Cov.	1548.850	2653.170	-	-	1	44	57		
	M12	X_1_ +	1710.406	4953.881	0.0187	0.0188	7	57	38		
		X_3_			0.0429	0.043					
IG(1, 0.1)	M13	Without Cov.	1583.957	3007.681	-	-	1	43	58		
	M14	X_1_ +	1805.174	7388.818	0.0187	0.0187	9	55	38		
		X_3_			0.0428	0.0429					
IG(0.1, 0.1)	M15	Without Cov.	1591.934	3295.849	-	-	1	43	58		
	M16	X_1_ +	1832.461	7814.488	0.0187	0.0187	9	55	38		
		X_3_			0.0426	0.0429					
IG(0.5, 0.05)	M17	Without Cov.	1595.658	3197.744	-	-	1	43	58		
	M18	X_1_ +	1780.736	6805.043	0.0187	0.0189	9	55	38		
		X_3_			0.0423	0.043					
IG(0.5, 0.0005)	M19	Without Cov.	1576.032	3424.368	-	-	1	44	57		
	M20	X_1_ +	1802.712	5995.237	0.0187	0.0188	8	56	38		
		X_3_			0.0429	0.043					

*G* = 5											
Hyperpriors		Models	DIC	WAIC	Posterior Quantities for Covariates		The number of areas per cluster				
					2.5 %	97.5 %	G1	G2	G3	G4	G5
IG(1, 0.01)	M21	Without Cov.	1719.839	5188.119	-	-	1	15	26	43	17
	M22	X_1_ +	1778.743	5295.266	0.0187	0.0188	1	11	32	38	20
		X_3_			0.0429	0.0435					
IG(1, 0.1)	M23	Without Cov.	1698.086	3771.668	-	-	1	15	26	43	17
	M24	X_1_ +	1703.022	3840.869	0.0187	0.0190	1	11	32	38	20
		X_3_			0.0427	0.0431					
IG(0.1, 0.1)	M25	Without Cov.	1880.830	8812.548	-	-	1	12	29	43	17
	M26	X_1_ +	**1567.252**	2918.976	0.0185	0.0188	1	11	32	38	20
		X_3_			0.0426	0.0432					
IG(0.5, 0.05)	M27	Without Cov.	1840.963	7065.505	-	-	1	12	29	43	17
	M28	X_1_ +	1738.459	5717.862	0.0187	0.0188	1	11	32	38	20
		X_3_			0.0429	0.0434					
IG(0.5, 0.0005)	M29	Without Cov.	1615.069	3337.529	-	-	1	15	26	43	17
	M30	X_1_ +	1596.172	**2726.028**	0.0186	0.0188	1	11	32	38	20
		X_3_			0.0426	0.0431					

The M1 model is an ST CAR localized model with *G* = 2 and an IG(1, 0.01) prior, without including covariates. The M2 model is also an ST CAR localized model with *G* = 2 and an IG(1, 0.01) prior, but it includes covariates X_1_ and X_3_. The M30 model, on the other hand, is an ST CAR localized model with *G* = 5 and an IG(0.5, 0.0005) prior, incorporating covariates X_1_ and X_3_. Based on the smallest DIC value after incorporating covariates, the Bayesian Spatio-temporal CAR localized model with *G* = 5 and a hyperprior IG(0.1, 0.1) (M26), was identified as the most appropriate model for estimating the relative risk (RR) of toddler stunting in Indonesia. However, according to the smallest WAIC value, the Bayesian Spatial CAR localized model with *G* = 5 and a hyperprior IG(0.5, 0.0005) (M30) was deemed more appropriate. Therefore, both models (M26 and M30) were run for comparison, and the results were found to be highly similar. For conciseness, we present the results from model M26 in the main manuscript, while the results for model M30 are provided as supplementary material. The summary results for parameters of the Bayesian spatio-temporal CAR localized model with *G* = 5 (M26) are presented in Table 5. The results showed that there was a significant positive association between increased poverty and LBW births and the risk of stunting.

**Table 5:** The summary of the posterior results for the parameters of the Bayesian spatio-temporal CAR localized model (M26).

	Mean	2.5 %	97.5 %	n.effective
Poverty	0.0186	0.0185	0.0188	1.5
LBW	0.0430	0.0426	0.0432	1.6
lambda1	−5.0093	−5.0560	−4.8518	3.6
lambda2	−1.0835	−1.1150	−1.0579	71.5
lambda3	−0.3548	−0.3717	−0.3457	73.7
lambda4	0.1253	0.1190	0.1301	73.3
lambda5	0.7024	0.6923	0.7080	90.8
delta	1.0166	1.0005	1.0623	2785.0
tau2	0.1016	0.0765	0.1349	24,022.4
rho.T	0.1207	0.0058	0.3146	24,293.4

LBW, low birth weight; lambda: the intercept for the appropriate cluster, where lambda1<lambda2< lambda3< lambda4< lambda5; tau2, the variance parameter associated with the spatial random effects ui; Delta, the penalty parameter; rho.T, the temporal autocorrelation parameter

**Fig. 1:**
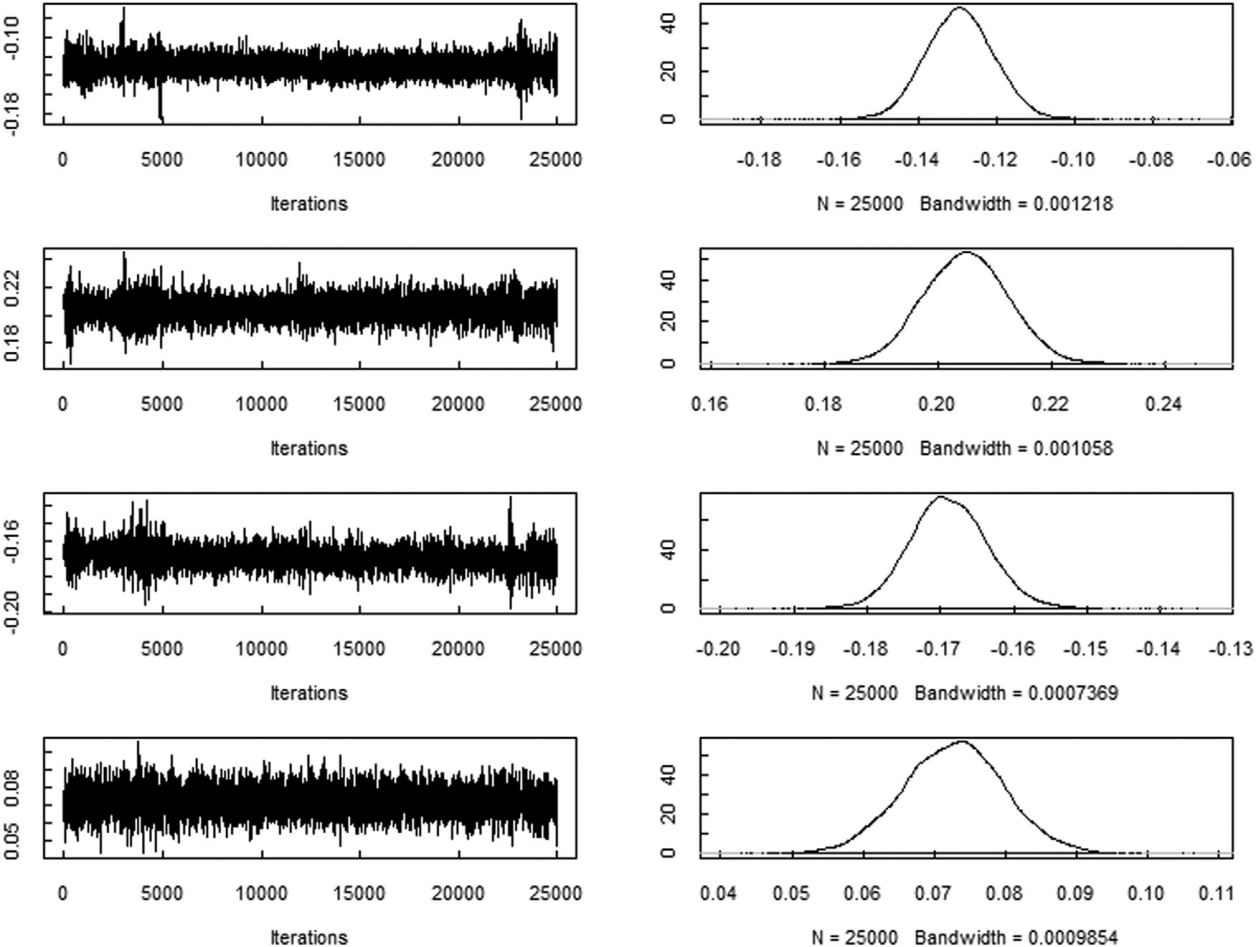
The trace plot of the spatial random effects parameters $u_{i}$ based on the prefered model (M26).

**Fig. 2:**
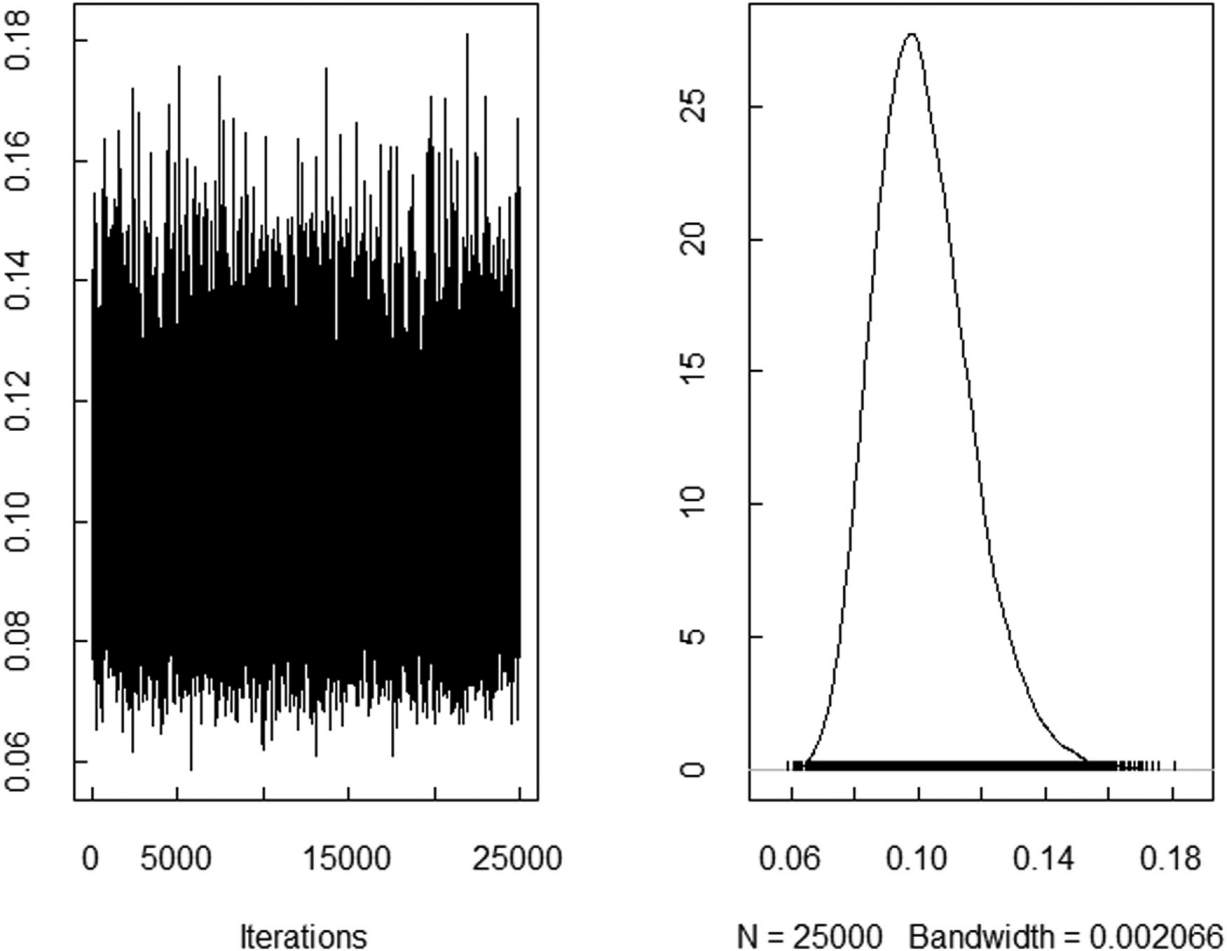
The trace plot of the variance terms $\tau^{2}$ based on the prefered model (M26).

The trace plots of the spatial random effects parameters $u_{i}$ and the variance terms $\tau^{2}$, based on the prefered model (M26), are presented in Fig. 1, Fig. 2, respectively, and indicates that the model has reached convergence.

The clustering of regions and RR of stunting cases in each province of Indonesia varied during 2020 to 2022 (Table 6).

**Table 6:** The localized structure (LS), RR stunting cases in Indonesia (2020–2022) in each district based on the preferred model (M26).

Areas	2020		2021		2022	
	LS	RR	LS	RR	LS	RR
Aceh	4	1.227	3	1.270	3	0.956
Bali	4	0.861	3	0.523	4	0.533
Bangka Belitung	2	0.302	3	0.625	3	0.467
Banten	3	0.449	3	0.702	4	0.817
Bengkulu	3	0.762	3	0.670	3	0.577
Gorontalo	3	1.009	3	0.899	3	0.895
DKI Jakarta	1	0.004	2	0.338	2	0.149
Jambi	3	0.695	2	0.317	3	0.486
Jawa Barat	4	0.825	4	0.872	4	0.821
Jawa Tengah	4	1.201	4	0.951	4	1.123
Jawa Timur	4	1.060	4	1.126	4	1.133
Kalimantan Barat	5	**2.519**	5	**2.215**	5	1.947
Kalimantan Selatan	4	1.202	5	1.094	4	1.116
Kalimantan Tengah	5	2.039	4	1.140	4	1.212
Kalimantan Timur	4	1.332	5	1.248	5	1.619
Kalimantan Utara	5	**2.637**	5	1.948	5	1.962
Kepulauan Riau	4	0.844	3	0.795	3	0.585
Lampung	2	0.487	3	0.642	3	0.540
Maluku	4	0.763	3	0.716	3	1.181
Maluku Utara	3	0.852	4	1.370	4	1.467
Nusa Tenggara Barat	5	1.853	5	**2.290**	5	**2.221**
Nusa Tenggara Timur	5	**2.421**	4	**2.384**	4	**2.676**
Papua	2	0.610	3	1.067	3	1.009
Papua Barat	3	1.220	3	1.388	4	1.516
Riau	3	0.707	3	0.635	3	0.498
Sulawesi Barat	5	2.084	5	2.038	5	**2.768**
Sulawesi Selatan	4	1.092	4	1.099	4	1.081
Sulawesi Tengah	4	1.260	4	1.394	4	1.571
Sulawesi Tenggara	4	1.536	4	1.945	4	1.321
Sulawesi Utara	3	0.489	2	0.311	2	0.276
Sumatera Barat	5	1.609	5	1.591	5	1.235
Sumatera Selatan	2	0.208	2	0.463	2	0.370
Sumatera Utara	3	0.627	4	0.706	3	0.658
DI Yogyakarta	4	1.095	4	1.112	4	1.097

**Bold:** three regions with the highest relative risk.

Table 6 shows that Nusa Tenggara Timur Province consistently ranks among the top three regions with the highest RR of stunting each year. In 2020, the highest RR values, according to the preferred model, were observed in Kalimantan Utara Province (RR = 2.637), followed by Kalimantan Barat Province (RR = 2.519) and Nusa Tenggara Timur Province (RR = 2.421). In 2021, the highest RR values were found in Nusa Tenggara Timur Province (RR = 2.384), followed by Nusa Tenggara Barat Province (RR = 2.290) and Kalimantan Barat Province (RR = 2.215). In 2022, the highest RR values were recorded in Sulawesi Barat Province (RR = 2.768), followed by Nusa Tenggara Timur Province (RR = 2.676) and Nusa Tenggara Barat Province (RR = 2.221).

**Fig. 3:**
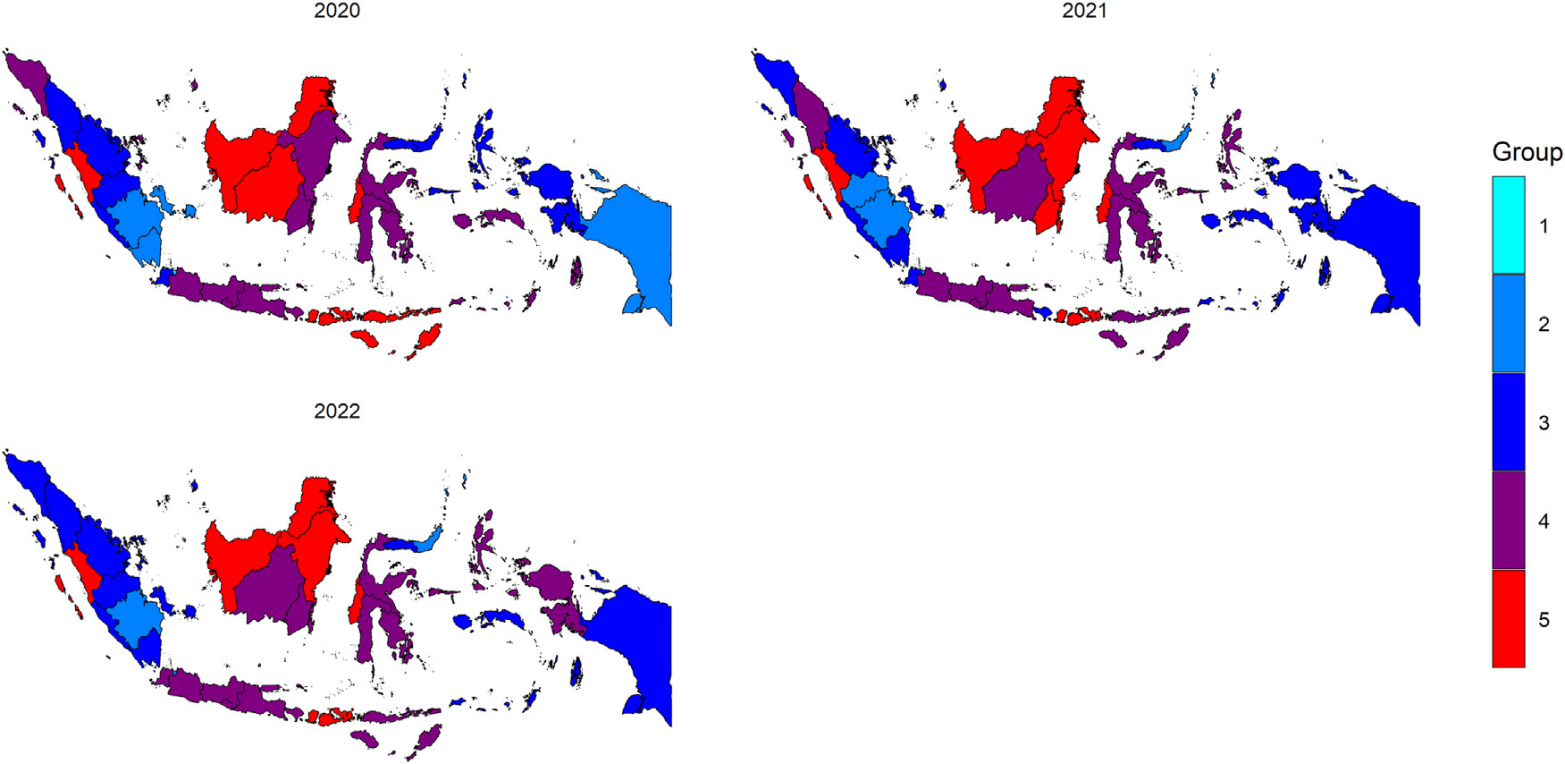
Thematic map of provincial Stunting case groupings in Indonesia from 2020 to 2022 based on the ST CAR localized *G* = 5 model with hyperprior IG(0.1, 0.1).

**Fig. 4:**
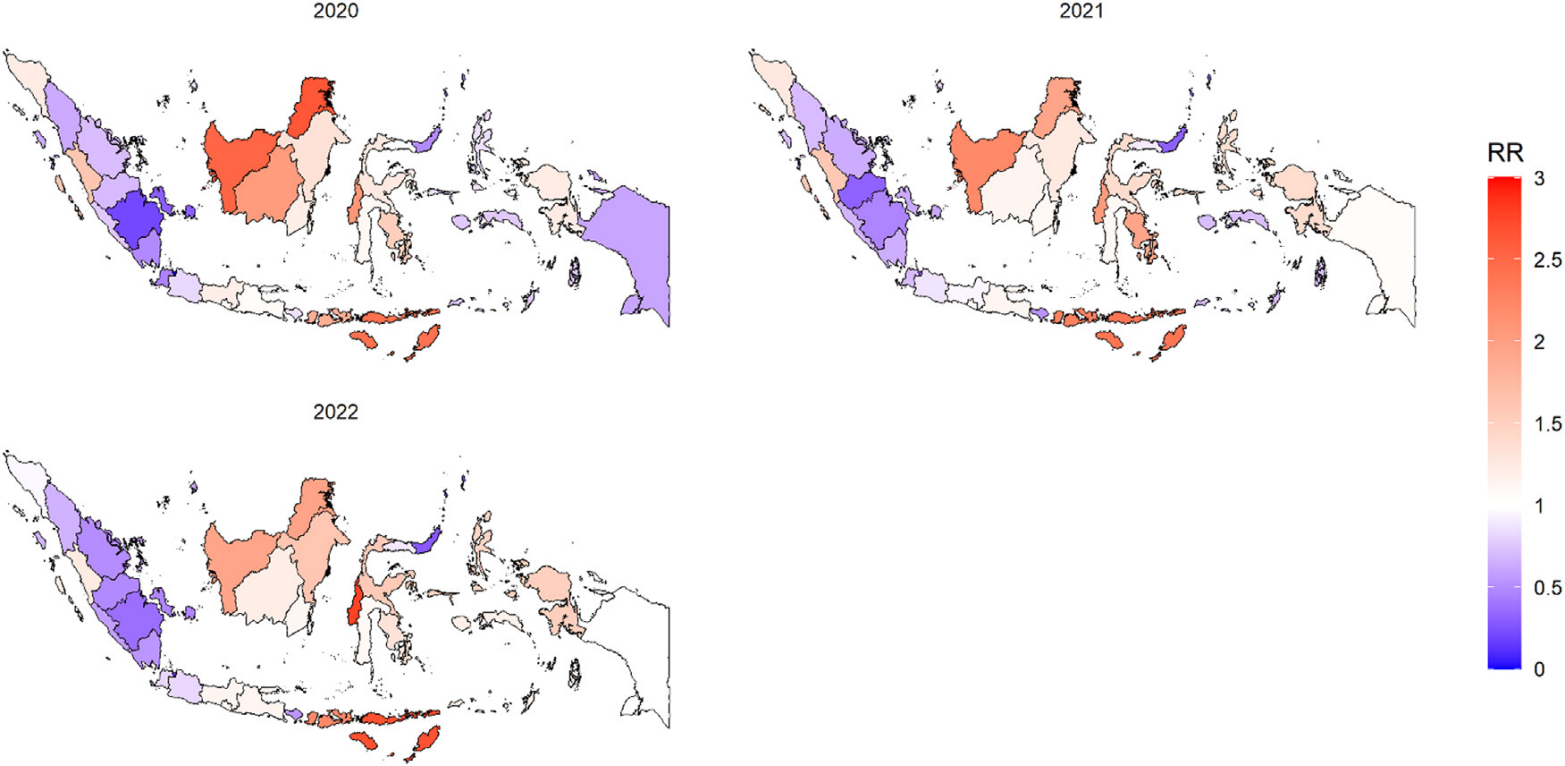
Mapping the RR of Stunting cases in Indonesia from 2020 to 2022 based on the localized *G* = 5 model with hyperprior IG(0.1, 0.1).

From Table 4, it is evident that the ST CAR localized model with *G* = 5, incorporating the percentage of poverty (X_1_) and the percentage of low birth weight (LBW) (X_3_), provides a better fit to the data compared to a corresponding model without covariates. The ST CAR localized model with *G* = 5, analyzed from 2020 to 2022, identified five groups (G1 = 1, G2 = 12, G3 = 29, G4 = 43, and G5 = 17) without covariates. When covariates were included, it also identified five groups, but with different numbers of areas in each group (G1 = 1, G2 = 11, G3 = 32, G4 = 38, and G5 = 20). Notably, one group (G1 = 1), consisting of a single district in one year (2020), was found in the DKI Jakarta province, which had the lowest RR (RR = 0.004) in 2020, as shown in Table 6. A thematic map depicting provincial stunting case groupings in Indonesia from 2020 to 2022, based on the ST CAR localized *G* = 5 model with hyperprior IG(0.1, 0.1), is presented in Fig. 3. Additionally, Fig. 4 maps the RR of stunting cases in Indonesia for the same period, utilizing the same localized *G* = 5 model and hyperprior.

## Conclusion and discussion

Several studies have investigated stunting and its determinants in Africa using Bayesian spatial modeling approaches [4,5], and Bayesian spatio-temporal approaches [2,3,9]. However, there has been no published application of the Bayesian spatio-temporal CAR model to address stunting cases in Indonesia. Existing models have not accounted for discontinuities in risk between neighboring areas. A more recent Bayesian ST CAR localized model allows for spatial autocorrelation among adjacent areas within discontinuous groups [21]. Such models are attractive because they capture the localized spatial structure of disease risks in complex urban areas, accommodating both smooth variations and abrupt changes (discontinuities) in values [26]. This paper introduces an application of the Bayesian ST CAR model to assess its effectiveness in modeling stunting cases and the influence of various associated covariates in Indonesia.

A total of 480 models were run: three variants of the Bayesian ST CAR localized model, each with five hyperpriors. These models explored 32 diverse combinations both with and without covariates. The Bayesian ST CAR localized model consistently identified poverty and recent low birth weight (LBW) as significant covariates. These findings partly corroborate previous research, which suggest that localized models are preferable for areas with high variability in count data [23,27]. The analysis revealed clustering of regions and RR of stunting across Indonesian provinces from 2020 to 2022. Nusa Tenggara Timur Province consistently ranked among the top three regions with the highest RR of stunting each year. The highest RR was observed in Sulawesi Barat Province (RR = 2.768), while DKI Jakarta exhibited the lowest RR (RR = 0.004) during the study period. Given the consistent identification of poverty and low birth weight (LBW) as significant predictors of stunting, targeted interventions are essential in high-risk provinces such as Nusa Tenggara Timur and Sulawesi Barat. Nutrition programs should prioritize pregnant women and young children, while maternal health services must be strengthened through improved antenatal care, enhanced maternal nutrition, and safe delivery practices. Poverty alleviation efforts such as financial assistance, job creation, and economic support for low-income families should also be emphasized to reduce nutritional deficiencies. Additionally, improving healthcare access by expanding facilities, training health workers, and implementing outreach services is vital for early detection and management of stunting in these high-RR regions. Specifically, areas identified with higher RR and grouped within high-risk clusters can be flagged for intensified public health efforts, while lower-risk clusters may require sustained or preventive strategies.

## Limitations and future works

Our findings are limited by the absence of comprehensive covariate data at finer spatial scales, such as districts or sub-districts, as well as by the lack of more recent data. A more up-to-date and complete dataset with additional variables would enhance the ability to capture trends and improve the timeliness and relevance of the findings. Furthermore, for the stunting data, the highest available temporal resolution was annual, which may impact the accuracy of group identification, and the relationships observed in the analysis. Future research should validate the model’s performance and conduct sensitivity analyses to assess how variations in spatial and temporal resolution influence the conclusions, a challenge commonly referred to as the modifiable areal unit problem (MAUP). Additionally, incorporating supplementary data sources such as satellite-derived environmental indicators, socioeconomic survey data, or geospatial health metrics may help address gaps in covariate information and enhance model accuracy. This study is grounded in real-world data obtained from the Directorate General of Regional Development, Ministry of Home Affairs, and the proposed methodology was directly applied to observed stunting cases across 34 provinces in Indonesia. By utilizing actual data, the analysis ensures practical relevance and policy applicability. However, we acknowledge that the absence of a simulation study limits the ability to fully assess the model’s performance under controlled conditions. Incorporating a simulation component in future research would allow for a more rigorous evaluation of the model’s accuracy, robustness, and sensitivity to different spatio-temporal structures, thereby enhancing the overall reliability and generalizability of the findings. Future research should explore the integration of heuristic and evolutionary optimization algorithms, such as the Gravitational Search Algorithm (GSA) and Inclined Planes System Optimization (IPSO), to enhance computational efficiency and predictive accuracy.

## Ethics statements

The authors confirm that this manuscript is an original work, has not been previously published, and is not under consideration for publication elsewhere. The authors declare no conflicts of interest related to this publication.

## CRediT authorship contribution statement

**Aswi Aswi:** Conceptualization, Methodology, Writing – original draft. **Septian Rahardiantoro:** Data curation, Visualization, Formal analysis, Investigation. **Anang Kurnia:** Supervision, Validation. **Bagus Sartono:** Supervision, Validation. **Dian Handayani:** Supervision, Validation. **Nurwan Nurwan:** Software, Visualization, Data curation.

## Declaration of competing interest

The authors declare that they have no known competing financial interests or personal relationships that could have appeared to influence the work reported in this paper*.*

## Data Availability

I have shared the link to my data

## Appendix Supplementary materials

Image, application 1
