# Supplementary material for: Bayesian spatio-temporal conditional autoregressive localized modeling techniques for socioeconomic factors and stunting in Indonesia
Source: MethodsX. 2025 Jun 24;15:103464. doi: 10.1016/j.mex.2025.103464 (PMC12268846; doi:10.1016/j.mex.2025.103464)
Supplement: Supplementary file 1 [file mmc1.docx]

Supplementary Table 1. The localized structure (LS), RR stunting cases in Indonesia (2020-2022) in each district based on the preferred model (M30)

| Areas | 2020 | | 2021 | | 2022 | |
| --- | --- | --- | --- | --- | --- | --- |
|  | LS | RR | LS | RR | LS | RR |
| Aceh | 4 | 1.228 | 3 | 1.271 | 3 | 0.957 |
| Bali | 4 | 0.861 | 3 | 0.524 | 4 | 0.533 |
| Bangka Belitung | 2 | 0.302 | 3 | 0.625 | 3 | 0.467 |
| Banten | 3 | 0.449 | 3 | 0.702 | 4 | 0.817 |
| Bengkulu | 3 | 0.763 | 3 | 0.670 | 3 | 0.577 |
| Gorontalo | 3 | 1.009 | 3 | 0.899 | 3 | 0.895 |
| DKI Jakarta | 1 | 0.004 | 2 | 0.339 | 2 | 0.149 |
| Jambi | 3 | 0.695 | 2 | 0.318 | 3 | 0.486 |
| Jawa Barat | 4 | 0.825 | 4 | 0.872 | 4 | 0.822 |
| Jawa Tengah | 4 | 1.201 | 4 | 0.951 | 4 | 1.123 |
| Jawa Timur | 4 | 1.061 | 4 | 1.126 | 4 | 1.133 |
| Kalimantan Barat | 5 | **2.514** | 5 | **2.209** | 5 | 1.942 |
| Kalimantan Selatan | 4 | 1.202 | 5 | 1.091 | 4 | 1.115 |
| Kalimantan Tengah | 5 | 2.035 | 4 | 1.140 | 4 | 1.212 |
| Kalimantan Timur | 4 | 1.331 | 5 | 1.246 | 5 | 1.617 |
| Kalimantan Utara | 5 | **2.632** | 5 | 1.946 | 5 | 1.959 |
| Kepulauan Riau | 4 | 0.845 | 3 | 0.795 | 3 | 0.585 |
| Lampung | 2 | 0.488 | 3 | 0.642 | 3 | 0.540 |
| Maluku | 4 | 0.763 | 3 | 0.716 | 3 | 1.180 |
| Maluku Utara | 3 | 0.852 | 4 | 1.369 | 4 | 1.467 |
| Nusa Tenggara Barat | 5 | 1.849 | 5 | **2.284** | 5 | **2.215** |
| Nusa Tenggara Timur | 5 | **2.413** | 4 | **2.383** | 4 | **2.676** |
| Papua | 2 | 0.612 | 3 | 1.067 | 3 | 1.009 |
| Papua Barat | 3 | 1.220 | 3 | 1.388 | 4 | 1.516 |
| Riau | 3 | 0.708 | 3 | 0.635 | 3 | 0.498 |
| Sulawesi Barat | 5 | 2.080 | 5 | 2.034 | 5 | **2.763** |
| Sulawesi Selatan | 4 | 1.092 | 4 | 1.099 | 4 | 1.081 |
| Sulawesi Tengah | 4 | 1.260 | 4 | 1.394 | 4 | 1.571 |
| Sulawesi Tenggara | 4 | 1.536 | 4 | 1.946 | 4 | 1.321 |
| Sulawesi Utara | 3 | 0.489 | 2 | 0.312 | 2 | 0.277 |
| Sumatera Barat | 5 | 1.607 | 5 | 1.589 | 5 | 1.233 |
| Sumatera Selatan | 2 | 0.209 | 2 | 0.464 | 2 | 0.371 |
| Sumatera Utara | 3 | 0.628 | 4 | 0.706 | 3 | 0.659 |
| DI Yogyakarta | 4 | 1.095 | 4 | 1.112 | 4 | 1.097 |
